# Experiences of Remote Provision across a Voluntary Sector Organisation Providing Mental Health and Wellbeing Services for Young People

**DOI:** 10.3390/ijerph20227086

**Published:** 2023-11-20

**Authors:** Joanne Worsley, Shaima Hassan, Lisa Nolan, Rhiannon Corcoran

**Affiliations:** 1 Department of Primary Care and Mental Health, University of Liverpool, Liverpool L69 7ZA, UK; s.m.hassan@liverpool.ac.uk (S.H.); corcoran@liverpool.ac.uk (R.C.); 2 NIHR Applied Research Collaboration North West Coast (ARC NWC), Liverpool L69 7ZA, UK; 3 NHS Cheshire and Merseyside Integrated Care Board, Liverpool L1 2SA, UK; lisa.nolan@liverpoolccg.nhs.uk

**Keywords:** remote provision, mental health, wellbeing, accessibility, therapeutic relationships, group programmes

## Abstract

The global COVID-19 pandemic presented not only challenges for services but also opportunities for reflection and change. This study aimed to understand young people, parents/carers, and professionals’ experiences of remote provision across a voluntary sector organization to inform the nature of future delivery. Reflections from professionals (*n* = 7), young people (*n* = 7), and parents/carers (*n* = 2) were collected through semi-structured interviews and focus groups. Data were thematically analysed. Five overarching themes were identified: ‘Accessibility’, ‘Remote therapeutic experiences’, ‘Translating to online’, ‘Spaces of comfort/discomfort’, and ‘Moving towards hybrid provision’. The COVID-19 pandemic changed service provision, notably with accelerated digitalisation. Although the service became more accessible, the digitalisation of services impacted the relational experiences for young people. Nevertheless, online provision was described as a ‘steppingstone’, allowing young people to engage in online therapy or group programmes before transferring to in-person provision. Although remote provision can lead to improvements in young people’s mental health, this format was not suitable for all. When considering future models of provision, assessing needs, preferences, and access to private space and hardware are all important considerations when deciding which format to use to achieve the best possible outcomes.

## 1. Introduction

Although the proportion of 6–16-year-olds with a probable mental health increased from one in nine (11.6%) to one in six (17.4%) between 2017 and 2021 [1], children and young people (CYP)’s mental health services are often poorly funded and typically characterised by long waiting lists [2]. Recent policy developments within the UK have, however, recognised the importance of prioritising CYP’s mental health [3,4].

Voluntary and community sector (VCS) counselling services for CYP—typically termed Youth Information, Advice, and Counselling Services (YIACS)—are well-placed to respond to the diverse range of needs that CYP are currently facing. The Future in Mind report [5] recognised the importance of YIACS, led by Youth Access, in providing early support for young people for a wide range of issues. The types of support available can vary across services, but typically include information, counselling, emotional support, access to health clinics, and advice and advocacy [6]. YIACS play an important role in engaging hard-to-reach and marginalised groups of young people, including young people from the LGBTQ+ and BME communities, as well as those with experience in the criminal justice system [7]. As well as achieving higher levels of satisfaction among young people, youth counselling provided by YIACS showed similar clinical outcomes to those reported in child and adolescent mental health services (CAMHS; [7]).

Although YIACS were traditionally delivered face-to-face, a number of youth mental health services, delivering counselling and support via online chat or videocall, operated before the global pandemic. Over recent years, there has been an increase in web-based therapies and support services [8]. Examples include text-based asynchronous communication (email), synchronous communication (instant messaging), and videoconferencing support [9].

According to one study exploring the effectiveness of an Australian online counselling service, the majority of young people accessing the service felt safer seeking support via online means, with many opting to engage in this way to reduce the emotional intensity of the conversation [10]. In line with this, a rapid evidence review of studies conducted within community-based mental health services demonstrated that remote interventions were found to be an effective way of supporting young people who find it difficult to access in-person provision (e.g., those living in remote locations) and can lead to positive outcomes among young people, including reductions in the severity of symptoms and increased wellbeing [11]. However, a number of challenges associated with remote provision were also highlighted in this review, such as disruptions due to poor signal, loss of non-verbal communication, and difficulties establishing a positive therapeutic alliance [11]. With regard to the latter, it is possible that the therapeutic process differs in this environment, as previous research suggested that practitioners spend more time building rapport online than accomplishing tasks [12]. Nevertheless, according to one study that investigated young people’s experiences of developing relationships with counsellors on Kooth.com (an online youth counselling service providing online counselling and support for young people aged 11–25 years in certain regions of the UK), therapeutic alliances sufficient to facilitate psychological change appeared possible online [13].

As VCS services innovatively responded to the global COVID-19 pandemic, developing new ways of working to support CYP’s mental health needs, the move to remote provision needs scrutiny in order to inform the nature of future delivery. The present study aimed to understand young people, parents/carers, and professionals’ experiences of remote provision across a VCS organisation in the North West of England. We were particularly interested in whether perspectives about remote provision were consistent or different across the three stakeholder groups (CYP, parents/carers, and professionals) and the implications for future practice. The purpose of this study was, therefore, to guide future service delivery so that remote provision can continue where it has been well-received but rethought or replaced where it has not.

## 2. Methods

### 2.1. Ethical Approval

Ethical approval was received from Preston Research Ethics Committee (IRAS ID: 297792).

### 2.2. VCS Organisation

The VCS service in question offers a wide range of support, wellbeing, and therapeutic services for CYP aged 5–25 years and their families [14], with CYP having the option to self-refer or access drop-in centres. In the face of COVID-19, the service developed a range of different ways of remotely supporting CYP, such as telephone or videoconferencing.

### 2.3. Participants

Posters advertising the study were circulated by a participation worker at the service to CYP, parents/carers, and professionals. All those who expressed an interest in participating were sent the participant information sheet and a blank copy of the consent form to complete and return to the research team via electronic means. The participant information sheet outlined the purpose of the study, potential benefits and risks from participation, and that participants would be renumerated for their time. A total of 16 participants took part: 7 young people (4 young people used the pronouns she/her, 1 young person used the pronouns he/him, and 1 young person used the pronouns they/them), 2 parents/carers, and 7 professionals such as counsellors, CYP wellbeing practitioners, and participation workers. With regard to CYP, six participants were 16 years or older, and one participant was under 16 years of age. Informed consent from a parent or legal guardian was obtained when required. All CYP were remunerated for their time.

### 2.4. Data Collection

Before data collection commenced, the first and second authors co-delivered presentations about the design and methods of the study to gain feedback from mental health participation workers and young people accessing community-based mental health services. Study materials, such as topic guides, were co-designed with CYP, a public advisor, a mental health promotion worker, and an assistant psychologist. All interviews and focus groups followed a semi-structured set of questions and were conducted via telephone and videocall. The topic guide comprised two sections focusing on the impact of COVID-19 on usual service provision and people’s experiences of using or delivering remote services. Data collection took place during February and April 2022. Interviews with staff members were conducted by the first author. Each interview lasted approximately 30 min. The first and second author co-facilitated three focus groups: one focus group comprised parents/carers, and two focus groups were conducted with CYP. During the focus groups, the researchers adopted a peripheral role by acting as ‘facilitators’ to encourage an interactive discussion. Each focus group lasted approximately one hour. CYP participated and responded to questions during the focus groups independently of parent/carer input. The interviews and focus groups were recorded and transcribed verbatim.

### 2.5. Analysis

As scientific independence from sociopolitical and personal attributes of researchers is critical, the team recognised the importance of remaining reflexive. Throughout the research process, the first author kept a reflexive diary. Contextual and reflexive notes, made during and following data collection, were documented in interview transcripts to facilitate the analysis.

The qualitative data was subjected to thematic analysis [15]. Thematic analysis is a qualitative method that aims to identify and report recurrent themes in data [15]. The six phases included familiarisation, generating initial codes, searching for themes, reviewing the themes, defining the themes, and naming the themes [15]. All transcription was undertaken by the first author to allow maximum data immersion. Line-by-line coding derived from a largely inductive approach was conducted by the first author to ensure that data were not overlooked. The codes applied remained close to participants’ own language and were reviewed by the second author. A wider research team was also consulted to consider alternate viewpoints. Codes were grouped together into categories, which were grouped into themes across stakeholder groups. Themes and subthemes were renamed and defined in consultation with the wider research team to ensure that the final analysis did not reflect the personal interpretation of one team member.

## 3. Results

Analyses of the focus group and interview data across the stakeholders revealed five overarching themes. A number of subthemes were identified in relation to each overarching theme (see Table 1).

### 3.1. Accessibility

#### 3.1.1. Heightened Accessibility for Certain Groups

In the face of COVID-19, the service developed a range of different ways of supporting CYP remotely, either via telephone or virtual means. Remote provision was perceived to be an effective way of supporting individuals who find it difficult to access in-person provision (e.g., inability to travel to attend sessions for financial reasons or diagnosis-related matters):

*It opens up a whole new realm of clients. For example, the agoraphobic, by definition they can’t leave the house*.

(Staff 03)

*I felt it made the service a lot more accessible to some young people and families. Thinking about [it] financially, if people are online, they’re not having to travel to a venue. If people have a lot of anxiety, then that might get in the way of therapy as you have to meet a new person or step into a new environment, having Zoom could be really beneficial for them clients*.

(Staff 06)

Following the easing of restrictions, group programmes returned to community hubs. Although the return to physical spaces was welcomed by many participants, one young person highlighted that this format excluded those who were unable to access in-person provision:

*[Group provision] has gone back in-person now, and I kind of miss it being online, because a lot of people can’t come to the face-to-face ones because of accessibility reasons*.

(CYP 02)

#### 3.1.2. Convenience

Online provision has been perceived as more convenient for CYP and their families, as this format removes the need to travel to a different location:

*For some young people, it’s made it more accessible than face-to-face work would. A young person might want to come to sessions but maybe the parents can’t be bothered with the commitment of having to take them every week after work*.

(Staff 06)

*What worked well? The accessibility is the main thing. If I wasn’t having a good day or whatever, it wouldn’t require so much effort for me to go out, even though I literally live down the road*.

(CYP 02)

Online provision also offers flexibility for parents/carers, especially those who have caring responsibilities for other young children:

*Some of the parents that I’ve worked with have other young children at home and trying to get a younger child out into another place, it’s awkward. So, it gives parents flexibility*.

(Staff 07)

*It was easy for me, because if I had to take him somewhere over lockdown, while my daughter is home… it’s like I’m in a state of panic then to try and get somewhere and to get back within that hour or two hours*.

(Parent 01)

Although restrictions have eased and the service has started to offer face-to-face appointments, many CYP have continued to opt to engage remotely, as this format is ‘convenient’. Thus, remote provision is a viable, and perhaps even preferred, option for some young people:

*We’re offering young people face-to-face, but most of our young people are still happy to go on Zoom because it’s very convenient*.

(Staff 03)

#### 3.1.3. Practical Issues for Accessibility

Remote provision enabled CYP to receive support from locations outside of the city region. For example, university students residing in the city centre whilst studying were able to access support outside of term-time, whereas other young people were able to attend therapy sessions whilst on vacation. Remote provision, therefore, facilitates continuation of care, which is an important consideration for CYP experiencing distress:

*I came to [name of city] for uni[versity] so I went back home to [name of town] a lot during the pandemic and I wouldn’t have been able to access those support services if they weren’t online*.

(CYP 02)

*Even if they’ve gone on holiday, young people could access it if they wanted to. I had one young man who still wanted to have the session and he was away in Wales and his parents made it accessible*.

(Staff 06)

Despite these advantages, participants acknowledged access barriers, with connectivity issues being a common area of difficulty for participants. Technological issues, such as problems with Wi-Fi, often impacted the quality of the call, with some young people reporting that their therapy sessions ended prematurely due to a poor signal:

*I know a couple of times when I was having therapy my Zoom cut out and I wasn’t able to finish the session. So just things like that made it a little more difficult*.

(CYP 02)

*I think sometimes the quality of a session could be affected just purely by technical issues*.

(Staff 07)

Concerns were also raised as remote provision was not accessible to those who do not have access to relevant hardware and/or Wi-Fi. To address this, young people now have the option to engage remotely whilst in school:

*Some young people may be living [in] relative situations of poverty. There are lots of children and families within this city that didn’t have access to a laptop, they didn’t have access to a private telephone [or] their own handset*.

(Staff 01)

*When you’re doing Zoom, you’ve got to think of the socioeconomics of people. Can they afford the kit to do it? But what I found is the ones that couldn’t afford it tended to do it from school, and schools have come on board pretty well*.

(Staff 03)

Although in many ways the service has become more accessible, many participants reported that the waiting list to access support has increased. Thus, the need for increased resources in the aftermath of the crisis to address the increase in child and adolescent mental health difficulties was highlighted:

*As we came out of the pandemic, we’ve seen an increase and our waits have been rising ever since*.

(Staff 05)

*Getting an appointment for your child is longer*.

(Parent 02)

#### 3.1.4. A Stepped Approach

Although the service operated an ‘open door’ policy where young people could walk into one of the community hubs to seek support (i.e., self-refer) prior to the pandemic, this method of entry may be too intimidating for certain individuals (e.g., those with social anxiety). In the face of COVID-19, the service developed a stronger social media presence, which improved access, as many young people reached out for support via these channels. In light of this, social media platforms were described as ‘a steppingstone for some young people’ (Staff 05) who feel confident using this these channels as a ‘first step’ to accessing support from the service:

*I think we have really developed our social media presence through COVID, seeing that as a bit of a front door to our service delivery… It’s been a steppingstone for some young people. So, some young people have reached out through our social media platforms and have now felt confident to be able to come in and see us*.

(Staff 05)

Online provision was also described as a ‘steppingstone’, allowing CYP to engage in therapy and develop a trusting relationship online with their therapist before transferring to in-person provision. This may be an important step for some CYP with mental health difficulties who find developing working relationships with others in-person difficult:

*I had a young lady who wouldn’t leave the house. She come on Zoom, and in the end, she did leave the house and came to one of the sessions*.

(Staff 03)

*I think it’s a really good steppingstone for those who are refusing school, just really bad social anxiety… I remember [name of CYP] said ‘I’m nervous about going to [the VCS service] to meet somebody. Could I have a Zoom session with them first?’ I think that’s really important because it was that virtual element that helped [name of CYP] ease [himself] into the counselling*.

(Staff 07)

Participants also perceived online group provision as a ‘steppingstone’, providing an opportunity for young people to meet similar others and build confidence in a ‘safer’ environment, which is an important step for those experiencing mental health issues who may find developing relationships with similar others in-person difficult:

*I might come across confident in these social spaces, but a couple years ago, I just had no confidence, and I wouldn’t have been able to do stuff. The fact that I can now is a large part because I was introduced to it online… So as much as I love in-person, and I prefer in-person, I think that there should be that online provision first*.

(CYP 02)

*We used to do specific groups so one for anxiety, self-harm groups [in-person]. The anxiety group, the attendance was really low because anxious people don’t want to go and sit in a room with people so I think if we were to get that up and running again, I think online would be really good as that steppingstone. So, they would all be on the screen together and then you can work towards actually all being in a room*.

(Staff 07)

### 3.2. Remote Therapeutic Experiences

#### 3.2.1. Drivers of Rapport

Therapeutic relationships constitute the essence of the therapeutic milieu (‘*The number one thing is the therapeutic alliance. If I don’t get that right, none of my skills work*’ (Staff 03)). Although professionals acknowledged that positive therapeutic relationships could be established via online means, young people reported difficulties forming trusting relationships online:

*Building up a relationship, warmth, laughter, humor could happen the same on Zoom… You’re still able to build a therapeutic relationship with children, young people and families*.

(Staff 06)

*I prefer face-to-face/in person because it is a lot more personal. You can really connect more with a person if you are with them*.

(CYP 04)

Professionals highlighted that online provision is useful insofar as it enables an insight into a young person’s home, which can aid the connection. For example, one therapist discovered a young person’s interest through observing a poster on her wall, which was then incorporated into future therapy sessions:

*Now that’s an advantage of Zoom over face-to-face because she wouldn’t have brought a poster with her into the [therapy] room. I saw that poster, and that led to some work where we took the characteristics from the superheroes that she likes, the ones that she’d like to be, and we helped her to become those characteristics*.

(Staff 03)

When compared to in-person provision, power imbalances were not as pronounced when engaging in online spaces, which may enable young people to feel more able to express themselves:

*I think it balances power, because suddenly I’m on a screen, so I’m a bit removed. So, this counsellor, because a lot of them think we’re the experts, and of course, we’re not. Certainly, for a person-centred point of view, we’re definitely not. I think it helps balance the power, because you’re a little face on the screen and they are a face on the screen. That’s what you are. Whereas when you’re in a room, there is a dynamic of you’re more the expert, or that’s what they can perceive*.

(Staff 03)

#### 3.2.2. Loss of Non-Verbal Cues

Despite these benefits, face-to-face provision was still favoured by many professionals, with one practitioner describing this format as ‘the best service model to have’ (Staff 05). In fact, therapy delivered via online means was perceived to be ‘less effective’ (Staff 03) as ‘the room presence’ (Staff 03) was lost:

*Zoom, it’s still not as good as face-to-face, but I would say probably 70% as good as face-to-face… I’ve lost 30%. In that 30% would be what I call the room presence… We’re human beings, we’re designed to be face-to-face. There’s a screen, you’re in a different place. I’ve lost about 30% in terms of nonverbal communication*.

(Staff 03)

In line with this, many professionals struggled to identify non-verbal cues during remote therapy sessions. Non-verbal communication is often incongruent with spoken words, and participants acknowledged that it is often harder to observe during remote sessions:

*I think you can pick up on what’s not being said a lot more when you’re sitting in a room with them, and you can pick up on the body language. So that’s why I found it difficult because they could just sit there on the screen and just go, ‘yeah, yeah, that’s fine, everything’s great’ when actually their hands could be fidgeting, they could be picking their nails, they could be scratching their legs*.

(Staff 07)

*The whole thing in general didn’t help me at all because she couldn’t tell when I was upset. She would just ask me the survey questions and then work on one thing, but I felt that I couldn’t really talk to her because it was online. I’d just answer but I wouldn’t go in depth about how I feel*.

(CYP 06)

### 3.3. Translating to Online

Therapeutic interventions and group programmes designed to be delivered face-to-face were translated into online formats in the face of COVID-19. With regard to individual therapy and support, therapists and practitioners struggled to recreate certain elements:

*He’s not good with the online… If he is doing a 1-1 on a Zoom, he gets bored very easy with it… But I think if you’re giving him that 1-1 [in-person] then he will do something with you because it’s something he enjoys doing… So, if you do an activity with him, a drawing or making something, I think you will get more out of him than you would through a Zoom*.

(Parent 02)

Service providers also highlighted a sense of loss in respect of the wider experiences surrounding group provision. For example, professionals struggled to recreate the important social aspects of in-person group provision. In an attempt to make online sessions more interactive and engaging, the service invested in tools, such as Kahoot (a game-based platform):

*We bring in online tools such as mentee.com, which allows everyone to have a say at the same time… There’s another one called Kahoot, and you can do fun quizzes and things on there. So, although it doesn’t feel as natural as face-to-face, we can try and incorporate other apps and bits of tech to try and increase that engagement. It’s not the same. And for people that do prefer face-to-face, you can’t always mitigate or recreate exactly the same circumstances, with exactly the same opportunities*.

(Staff 01)

Despite such efforts, many professionals and young people favoured being together in a shared physical space (‘*I definitely preferred groups pre-COVID*’ (CYP 01)), as in-person group provision felt less ‘structured’, allowing natural peer conversations to occur. As young people had previously found value in the surrounding social opportunities available during in-person provision, physical spaces were found to socially enrich group work in a way that was lost when connecting remotely. As a result, there was a sense amongst participants that online group provision offered a pale imitation of what had previously been available in-person:

*I think in-person groups work better, because of how you can utilise the space. It allows young people to have those informal conversations and utilise that as a social space, where the online groups… they’re a bit more structured, it’s a little bit harder to have those informal discussions. It’s harder to establish relationships, particularly if you’re trying to get young people into groups to reduce isolation… For that cohort of young people, LGBTQ, coming to a venue and that to be their safe space to be able to express themselves how they would like to express themselves is really important, and that was lost through the pandemic*.

(Staff 05)

*Making connections outside the group was more difficult… There’s not really that time to just talk to people because the people who are running the sessions are leading it, you can’t just go in breakout rooms and have a chat with your friend*.

(CYP 02)

Some young people found the group sessions harder to navigate online, as they noticed themselves frequently talking over others. Online platforms, such as Zoom, have a ‘hands-up’ function, which offered a potential solution to this; however, using this feature was found to disrupt the natural flow of conversation:

*You’ll lose the natural flow of conversation sometimes… When you’re working with groups of young people, if you were in a physical space, you could have six people over there… There can be lots of that informal, non-structured or even semi-structured time where you may have multiple and really natural conversation and warmth happening and relationships developing. Whereas when you’re in an online framework, it’s very structured as people have to use the hands up function before they speak*.

(Staff 01)

*I think using the hands up feature is great, but it can also stop it from being like a flowing conversation sometimes*.

(CYP 03)

As a result, there was an increase in individual therapeutic work with LGBTQ+ young people, as many did not feel comfortable engaging in group provision online:

*For our LGBTQ project, we’ve seen an increase of one-to-one work during the pandemic, and that’s because a lot of them didn’t feel comfortable accessing a group online. Now we’re returning back to face-to-face that has started to decrease slightly, because we’re able to hold them in the group space… Those groups actually have a really meaningful role to play in supporting children’s and young people’s positive mental health… They are often seen as early intervention/prevention, but the value of them can’t be underestimated*.

(Staff 05)

### 3.4. Spaces of Comfort/Discomfort

Engaging in therapy from the home environment was beneficial for some young people, as they are often ‘*so used to being on a screen*’ (Staff 07) and are engaging from ‘*their own comfort zone*’ (Staff 03), which enabled them to feel more able and willing to discuss difficult experiences and/or abstract feelings:

*You’re in their environment and it is unique in that respect, because obviously when they come to the centre, it’s all quite clinical… There they are in their own comfort zone*.

(Staff 03)

*For young people, it could potentially be easier because young people in this generation are so used to being on a screen. I think they find it a lot easier than having to go and sit in a room with somebody. There’s a lot less pressure to present yourself a certain way if you’re on Zoom… I think for some, it offers a level of protection and reassurance*.

(Staff 07)

When engaging from their home environment, young people were surrounded by their family members, and this was beneficial for some. For example, one young person was allowed to have her younger sibling attend her therapy sessions, which ‘*calmed her brilliantly*’ (Staff 03):

*There was one when a little brother came in and that was quite interesting because it was a female client and she said, ‘do you mind if he sits with me’ because she was a bit nervous… He came in and it calmed her brilliantly*.

(Staff 03)

In contrast, many professionals highlighted that lack of access to a safe and private space within the home environment hindered engagement. For some young people, this environment was the context where they had experienced difficulties, which made it harder for them to disclose and articulate their emotions:

*Some children or young people just don’t feel safe. If we think about practicality, there are some children and young people who don’t have a private space at home, where they could reasonably or safely discuss the things that were impacting them. So, some young people may have shared a bedroom, could have lived in a quite chaotic household, may not have had a private space to have those conversations*.

(Staff 01)

*I think for young people, particularly those who experience family issues, it can be really difficult to engage from home. So, for example, there was a young person who actually declined the online support, because [of]… the issues that he was presenting with which were home related family issues. So, it was difficult for them to work with that within the environment that he struggles in*.

(Staff 02)

Young people perceived online provision as an invasion of their privacy, as family members could overhear or other young people and professionals could see their private space:

*I didn’t access it in my house. I could have accessed it in my house, but I didn’t want to mainly because of people hearing… In my house it would be weird because I have siblings who could barge in*.

(CYP 07)

### 3.5. Moving towards Hybrid Provision

Participants highlighted the importance of offering remote provision alongside face-to-face support. As it became apparent that ‘*one size will never fit all*’ (Staff 01), the online offer should be embedded in future service delivery. In order to build a service that is person-centred, it is important to offer CYP and their families a choice:

*Every child, young person and family are really individual. So, it’s really about considering how one size will never fit all*.

(Staff 01)

*I think we need to treat them as individuals, and not a one size fits all. We need to go on a basis of that person, that child, and what is going to be best for them*.

(Parent 01)

As online provision remained vital for many CYP, service providers explored creative means of integrating online and in-person provision, affording young people the option of attending a session either in-person or remotely:

*I’m trying to do blended sessions now… We’ve got the ability to put on a big screen young people in a Zoom space, while others are in a face-to-face space*.

(Staff 01)

Young people also emphasised the importance of adopting a blended approach, so everyone has the option to attend each session either in-person or via online means:

*I’d be interested to do an in-person session but have online with it, so [name of CYP] could still be there if we were all there in-person, [name of CYP] could still be there on Zoom with us so she is still there but the people who want to be in-person can*.

(CYP 04)

*[Name of group] has gone back in person now, and I kind of miss it being online, because a lot of people can’t come to the face-to-face ones because of accessibility reasons. So again, it’s a shame that they got rid of the online one to replace it with face-to-face. I feel like there should be both*.

(CYP 02)

## 4. Discussion

This study set out to understand young people, parents/carers, and professionals’ experiences of remote provision across a voluntary sector organisation in the North West of England. In the face of COVID-19, the service developed a range of different ways of supporting people remotely, either through telephone or virtual means. The findings across the three stakeholder groups were generally consistent; however, the minimal differences are outlined below.

Consistent with previous evidence (e.g., [11]), remote provision was perceived to be an effective way of supporting young people who find it difficult to access in-person provision (e.g., inability to travel to attend sessions for financial reasons or diagnosis-related matters). While the findings suggested that remote provision can reduce barriers to access for many young people, practitioners further acknowledged that this format also poses barriers and exacerbates inequalities for others.

Although the service operated an ‘open door’ policy prior to the pandemic, where young people could walk into one of the community hubs to seek support (i.e., self-refer), practitioners suggested that this method of entry may be too intimidating for certain individuals (e.g., those with social anxiety). In the face of COVID-19, the service developed a stronger social media presence, which improved access, as many young people reached out for support via these channels. In light of this, practitioners described social media platforms as ‘steppingstones’. Online provision was also described as a ‘steppingstone’ by practitioners, as this format offered CYP an opportunity to engage in therapeutic interventions or group programmes via online means before having the option to transfer to in-person provision. This presents an opportunity for CYP to familiarise themselves with the format of the sessions before accessing the service, which may be an important first step for those with mental health difficulties. The re-use of the notion ‘steppingstone’ further highlights the variety of ways in which the adaptations implemented in the face of the COVID-19 pandemic are advantageous additions to service as usual. Consistent with previous research [10], many young people felt safer engaging via online means. In light of this, it is crucial to retain online provision for certain groups, such as those with physical or mental health conditions, who may find engaging with support in-person challenging.

While our findings suggested that therapeutic alliances sufficient to facilitate psychological change appear possible online [13], practitioners acknowledged that it often takes longer to establish these relationships whilst working remotely (e.g., [12,16]). Both professionals and CYP emphasised a sense of loss in respect of the personal connection, as non-verbal cues are harder to identify in this milieu. Consistent with previous research (e.g., [16]), practitioners also highlighted a sense of loss in respect of the wider experiences surrounding group provision. In particular, service providers struggled to recreate the social aspects of in-person group provision [16], and participants found group sessions online to be more structured, which resulted in a number of LGBTQ+ young people seeking individual support from the service. Although these spaces were important for young people during periods of lockdown, group programmes returned to physical spaces following the easing of restrictions, which then excluded those who face physical or psychological barriers (e.g., inability to travel to attend sessions for financial reasons or diagnosis-related matters).

These findings have implications for VCS mental health services worldwide. First, in relation to the mode of delivery, our findings suggested that a ‘one size fits all’ strategy may not be appropriate following the COVID-19 pandemic, as some CYP preferred engaging via remote means whilst others were keen to engage in-person. In order to build a service that is person-centred, remote provision should complement or co-exist alongside face-to-face provision, providing CYP and their families a choice of formats. According to practitioners, remote provision is less effective than its in-person counterpart, with the latter referred to as the ‘best service model’. Exploring the effectiveness of each form of provision is important for both CYP and practitioners, as it is important for the format to be effective for those delivering the service as well as for the CYP accessing the service. Furthermore, similar VCS services should consider developing a stronger social media presence, as many young people utilised these platforms to reach out for support during the pandemic. Last, if VCS services are to continue operating remotely, investing in interactive tools, such as Kahoot (a game-based platform), to enhance engagement during group provision should be encouraged.

The reported research has limitations. First, only two parents/carers participated in this study. Further research is, therefore, required, as the inclusion of a broader sample of parents/carers would have resulted in a greater understanding of experiences. Similarly, although the VCS service in question supports children as young as five years, the majority of the CYP who participated in our study were 16 years or older. Future research should, therefore, incorporate the voices of younger children. As data collection took place via online platforms, our sample may represent those who are engaging well with remote provision. Thus, these findings are limited and may not be representative of the views held by all young people and parents/carers. Nevertheless, mixed views on remote provision were elicited across all stakeholder groups. If VCS services are to continue the remote offer, comparative studies investigating the outcomes of remote versus in-person provision should be prioritised.

## 5. Conclusions

Overall, alternative modes of provision are regarded as advantageous additions to service as usual. The changes catalysed by the COVID-19 pandemic resulted in the VCS service becoming more accessible; however, this format was not suitable for all. When considering future models of provision, assessing needs, preferences, and access to private space and hardware are all important considerations when deciding which format to use to achieve the best possible outcomes.

## Figures and Tables

**Table 1:** Overarching themes and subthemes.

Themes	Subthemes
Accessibility	Heightened accessibility for certain groups
	Convenience
	Practical issues for accessibility
	A stepped approach
Remote therapeutic experiences	Drivers of rapport
	Loss of non-verbal cues
Translating to online	
Spaces of comfort/discomfort	
Moving towards hybrid provision	

## Data Availability

Qualitative data extracts are presented in the article to support the findings. The data generated and analysed during the current study are not publicly available, as the data collected is sensitive and could compromise the confidentiality and anonymity of the participants, but are available (limited) from the corresponding author on reasonable request.

## References

[1] Newlove-Delgado T., Williams T., Robertson K., McManus S., Sadler K., Vizard T., Cartwright C., Mathews F., Norman S., Marcheselli F. (2021). Mental Health of Children and Young People in England, 2021.

[2] Peytrignet S., Marszalek K., Grimm F., Thorlby R., Wagstaff T. (2022). Children and Young People’s Mental Health: COVID-19 and the Road Ahead.

[3] Five Year Forward View for Mental Health 2016. https://www.england.nhs.uk/wp-content/uploads/2016/02/Mental-Health-Taskforce-FYFV-final.pdf.

[4] NHS England (2019). NHS Long Term Plan. https://www.longtermplan.nhs.uk/wp-content/uploads/2019/08/nhs-long-term-plan-version-1.2.pdf.

[5] Department of Health and NHS England (2015). Future in Mind: Promoting, Protecting and Improving Our Children and Young People’s Mental Health and Wellbeing. https://assets.publishing.service.gov.uk/government/uploads/system/uploads/attachment_data/file/414024/Childrens_Mental_Health.pdf.

[6] Youth Access (2017). The YIACS Model. http://www.youthaccess.org.uk/about-us/the-yiacs-model.

[7] Duncan C., Rayment B., Kenrick J., Cooper M. (2020). Counselling for young people and young adults in the voluntary and community sector: An overview of the demographic profile of clients and outcomes. Psychol. Psychother. Theory Res. Pract..

[8] Hanley T., Prescott J., Sefi A. (2022). The therapeutic goals set by university students in an anonymous web-based therapy and support setting. Front. Psychol..

[9] Barak A., Klein B., Proudfoot J.G. (2009). Defining internet-supported therapeutic interventions. Ann. Behav. Med..

[10] Navarro P., Bambling M., Sheffield J., Edirippulige S. (2019). Exploring young people’s perceptions of the effectiveness of text-based online counseling: Mixed methods pilot study. JMIR Ment. Health.

[11] James K. (2020). Remote Mental Health Interventions for Young People: A Rapid Review of the Evidence.

[12] Williams R., Bambling M., King R., Abbott Q. (2009). In-session processes in online counselling with young people: An exploratory approach. Couns. Psychother. Res..

[13] Hanley T. (2012). Understanding the online therapeutic alliance through the eyes of adolescent service users. Couns. Psychother. Res..

[14] Hassan S.M., Worsley J., Nolan L., Fearon N., Ring A., Shelton J., McEgan D., Yameen F., Khedmati E.M., Kullu C. (2022). An exploration of young people’s, parent/carers’, and professionals’ experiences of a voluntary sector organisation operating a Youth Information, Advice, and Counselling (YIAC) model in a disadvantaged area. BMC Health Serv. Res..

[15] Braun V., Clarke V. (2006). Using thematic analysis in psychology. Qual. Res. Psychol..

[16] Worsley J., Hassan S., Nolan L., Corcoran R. (2022). ‘Space to hide’: Experiences of remote provision across child and adolescent mental health services (CAMHS). BMC Health Serv. Res..

